# Repurposing Colchicine in Treating Patients with COVID-19: A Systematic Review and Meta-Analysis

**DOI:** 10.3390/life11080864

**Published:** 2021-08-23

**Authors:** Chi-Hone Lien, Ming-Dar Lee, Shun-Long Weng, Chao-Hsu Lin, Lawrence Yu-Min Liu, Yu-Lin Tai, Wei-Te Lei, Jui-Ming Liu, Ya-Ning Huang, Hsin Chi, Nan-Chang Chiu, Chien-Yu Lin

**Affiliations:** 1 Hsinchu MacKay Memorial Hospital, Hsinchu 30071, Taiwan; 4976@mmh.org.tw (C.-H.L.); 4554@mmh.org.tw (M.-D.L.); 4467@mmh.org.tw (S.-L.W.); 3099@mmh.org.tw (C.-H.L.); drlawrenceliu@gmail.com (L.Y.-M.L.); superlof@gmail.com (Y.-L.T.); lazyleisure@gmail.com (W.-T.L.); 2 Department of Medicine, MacKay Medicine College, New Taipei City 25160, Taiwan; chi.4531@mmh.org.tw (H.C.); ncc88@mmh.org.tw (N.-C.C.); 3 Department of Biological Science and Technology, National Chiao-Tung University, Hsinchu 30010, Taiwan; 4 Graduate Institute of Clinical Medical Sciences, College of Medicine, Chang Gung University, Taoyuan 33302, Taiwan; 5 Department of Urology, Taoyuan General Hospital, Ministry of Health and Welfare, Taoyuan 33004, Taiwan; mento1218@gmail.com; 6 Department of Pediatrics, MacKay Children’s Hospital, Taipei 10449, Taiwan; yvon1207@gmail.com

**Keywords:** COVID-19, novel coronavirus, SARS-CoV-2, colchicine, immunomodulation

## Abstract

Coronavirus disease 2019 (COVID-19) had caused huge health losses worldwide. Several drugs had been applied to treat patients with COVID-19, and repurposing colchicine had been proposed for its anti-inflammatory properties via several pathways. In this systematic review, we evaluated the effects of colchicine treatment. From inception to May 31, 2021, databases, including PubMed, EMbase, medRxiv, and Research Square were searched, and 11 studies were enrolled. A total of 17,205 COVID-19 patients with male predominance (62.9%) were analyzed. Patients with colchicine treatment had a significantly lower risk of mortality (odds ratio (OR): 0.57, 95% confidence interval (CI): 0.38–0.87, *I*^2^: 72%; *p* < 0.01) and a non-significantly lower rate of mechanical ventilation (OR: 0.67, 95%CI: 0.39–1.15). The side effects were mild and not significantly different (OR: 2.03, 95%CI: 0.51–8.09). Subgroup analysis with randomized controlled trials showed no statistically significant difference in the mortality (OR: 0.80, 95%CI: 0.44–1.46, *I*^2^: 33%; *p* = 0.22). In conclusion, our meta-analysis found that colchicine treatment was associated with a significantly lower risk of mortality in patients with COVID-19. However, this benefit was not observed in the subgroup analysis of randomized controlled trials. Further randomized controlled studies are required to confirm the potential benefits of colchicine treatment.

## 1. Introduction

The emerging crisis of the coronavirus disease 2019 (COVID-19) caused by severe acute respiratory syndrome coronavirus 2 (SARS-CoV-2) had caused huge healthy loss worldwide [1,2,3]. As of 31 May, 2021, there were more than 170 million patients infected, and the mortality rate was approximately 2% [1]. Immune-mediated inflammatory processes played a crucial role in the pathophysiology of COVID-19, and pleiotropic cytokine alterations had been observed in patients with COVID-19 [4,5,6]. In patients with severe COVID-19, higher interleukin (IL)-6, IL-10, granulocyte-colony stimulating factor (G-CSF), monocyte chemoattractant protein 1 (MCP1), macrophage inflammatory protein (MIP)1α, and tumor necrosis factor (TNF)-α were reported. Overt cytokine storm will cause disseminated systemic inflammation and progress to acute respiratory failure or disseminated intravascular coagulation [5]. In conclusion, immune-mediated inflammatory responses were believed to involve in the pathophysiology of severe COVID-19.

The optimal treatment against COVID-19 is still under investigation [7]. There are some antiviral agents used to combat COVID-19, such as remdesivir. In patients requiring oxygen but no mechanical ventilation, remdesivir treatment is associated with a better outcome [8]. Since cytokine alterations are overt in COVID-19, immunomodulatory agents may be beneficial in treating patients with severe COVID-19 in addition to antiviral medication. Corticosteroids have strong anti-inflammatory effects, and patients receiving dexamethasone have better clinical outcomes [9]. IL-6 inhibitor and Janus kinase inhibitors may also improve the clinical outcomes of COVID-19. However, not all drugs are effective and may be harmful, such as hydroxychloroquine. The best medical strategies to combat COVID-19 remain largely unclear.

Colchicine is an old drug derived from autumn crocus (*Colchicum autumnale*) and is commonly used to treat gout, Behçet’s disease, and familial Mediterranean fever [10]. It prevents microtubule assembly and leads to subsequent disrupts of multiple inflammatory pathways, including NOD-, LRR- and pyrin domain-containing protein 3 (NLRP3) inflammasome activation, microtubule-based inflammatory cell chemotaxis, pore formation activated by purinergic receptors P2X7 and P2X2, generation of leukotrienes and cytokines, and phagocytosis [11]. Physicians are familiar with its usage, and it is cheap, easily available, and accessible. The adverse events are mainly gastrointestinal and usually mild and tolerable [12]. Based on the immune-mediated properties of COVID-19 infection and the immunomodulatory effects of colchicine, repurposing the use of colchicine has been applied in the COVID-19 pandemic [13]. Reduced lung injury by colchicine use has been reported in rats with acute respiratory syndrome [14]. During the pandemic, colchicine treatment is associated with better outcomes in previous studies [15]. However, not all studies have the same finding [16,17]. Therefore, we conducted this systematic review and meta-analysis to investigate the effects of colchicine in treating patients with COVID-19.

## 2. Materials and Methods

### 2.1. Study Design and Literature Search

Our study was approved by the Institutional Review Board of the MacKay Memorial Hospital, Taipei, Taiwan (approval number, 20MMHIS140e) and conducted in accordance with the Preferred Reporting Items for Systematic Reviews and Meta-analyses (PRISMA) guideline [18,19]. This trial was registered in PROSPERO with registry number CRD42021270201. We used comprehensive keywords, such as “COVID-19”, “COVID-2019”, “severe acute respiratory syndrome coronavirus 2”, “2019-nCoV”, “2019nCoV”, “SARS-CoV-2”, and “Wuhan” with Boolean operators and MeSH terms. The complete search strategy was attached as Supplementary File S1. Electronic medical databases were searched from inception to 31 May 2021, including PubMed/Medline, EMBASE, Art Image Indexing Service on the Internet Database (Chinese database), and the Cochrane database. Preprint medical databases were also searched, including medRxiv and Research Square. The search was independently performed by two authors, and disagreements were resolved through a discussion with the third author. No constraints were placed on language, year of publication, and participant characteristics to ensure a comprehensive search and identify the maximum number of potential articles.

### 2.2. Study Selection and Data Extraction

Randomized controlled studies or cohort studies investigating “colchicine”, “immunomodulation”, or “anti-inflammation” on COVID-19 were analyzed. The exclusion criteria were as follows: duplicate publications, irrelevant articles, studies where the infection status was not clearly confirmed, studies that did not evaluate clinical outcomes, simple case reports, and review articles. Primary outcomes were the effects of colchicine on mortality. Secondary outcomes were the effects of mechanical ventilation and adverse events.

Furthermore, two authors independently appraised the selected articles and extracted the following data: name of the first author, study country, participant population, demographic data, dosage and duration of colchicine, concomitant medication, clinical outcomes, adverse events, and author conclusion. For quality assessment, we used the revised Cochrane risk-of-bias tool for randomized trials (RoB 2) for randomized controlled trials and Newcastle-Ottawa Scale (NOS) for observational cohort studies [20,21]. Quality assessments were conducted independently by two authors based on the domains of selection, ascertainment, causality, and reporting [22]. In case of disagreement between the two authors, a consensus was reached through a discussion with the third author.

### 2.3. Statistical Analyses

Reported odds ratios of enrolled studies were pooled to calculate the odds ratio (OR) of colchicine treatment on mortality, mechanical ventilation, and adverse events. If meta-analysis was performed, a random-effect regression model was used, assuming that the true effect size was not the same. Heterogeneity was further quantified using Cochran’s Q test and *I*^2^ statistics. The heterogeneity was considered low, moderate, and high for *I*^2^ < 50%, 50% to 75%, and >75%, respectively [22]. Sensitivity analysis was conducted to investigate the impact of individual studies. Potential small study bias was evaluated by funnel plots and by Egger’s regression test [23]. A *p*-value less than 0.05 was considered statistically significant. We also performed several predefined subgroup analyses to determine if the pooled odd ratios were affected by some factors, including mortality rates, study types, and study population. MedCalc (MedCalc Software, Ostend, Belgium) v18 and R software version 4.0.3 (R Foundation for Statistical Computing, Vienna, Austria) were used for statistical analyses.

## 3. Results

### 3.1. Enrolled Studies and Demographic Characteristics

**Figure 1:**
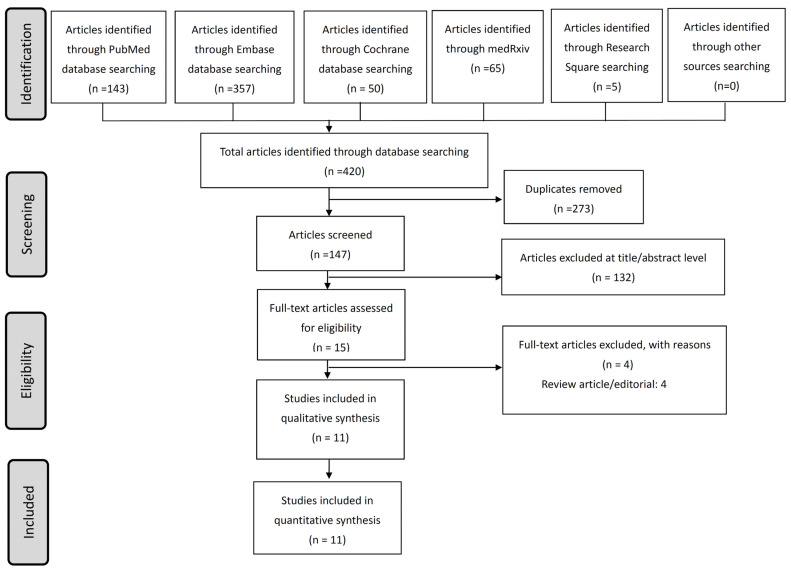
Flowchart of literature search and enrolled studies.

As of 31 May 2021, 147 non-duplicated articles were selected from the medical research database (Figure 1). The titles and abstracts of all articles were screened, and 11 studies fulfilling the inclusion and exclusion criteria were included in the final systematic review (Table 1) [16,17,24,25,26,27,28,29,30,31,32]. Four studies were randomized controlled trials, and the others were observational cohort studies. Two randomized controlled trials involved multiple countries and four studies in Europe, three in the USA, two in Brazil, two in Colombia, and one in India. A total of 17,205 participants with male predominance (62.9%) were identified, and 10 studies recruited hospitalized patients. A wide range of mortality rates was reported from 0% to 72.9%. Authors of seven studies supported the benefits of colchicine; two studies had marginal benefits, one study showed no significant difference, and one study had no comment.

**Figure 2:**
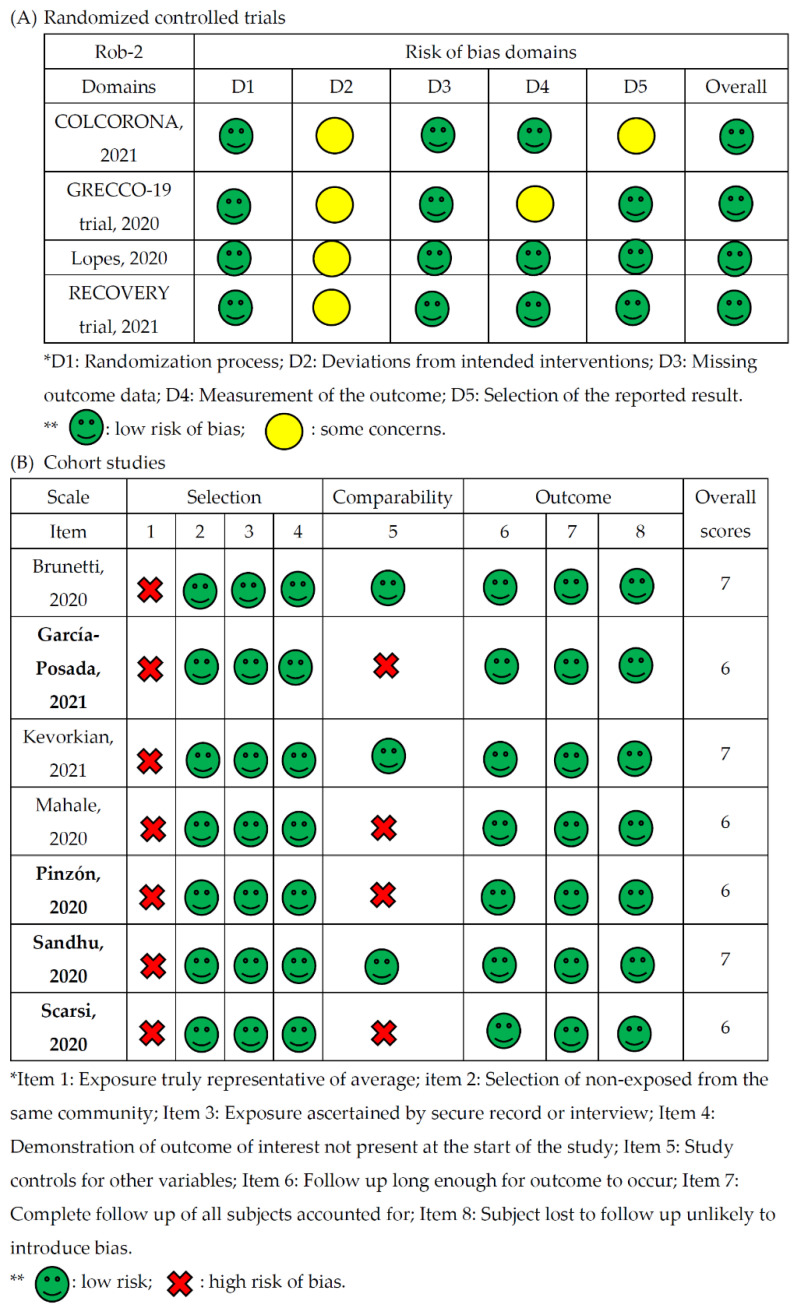
Quality and risk assessment of enrolled studies.

Quality assessments were conducted and summarized in Figure 2. Most studies reported low bias, and the quality of studies was rated suitable. All studies were qualified to be enrolled in further meta-analysis.

### 3.2. Meta-Analysis of Colchicine Treatment on Mortality

**Figure 3:**
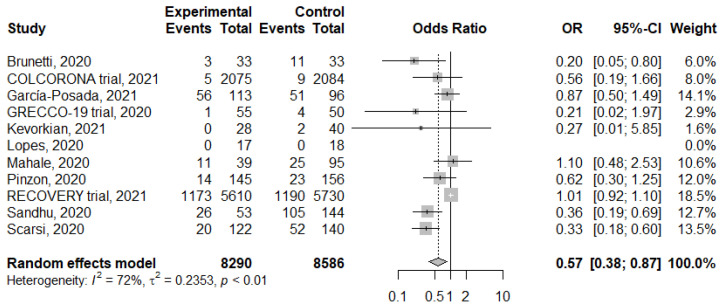
Forrest plot of mortality in colchicine group and control group.

**Figure 4:**
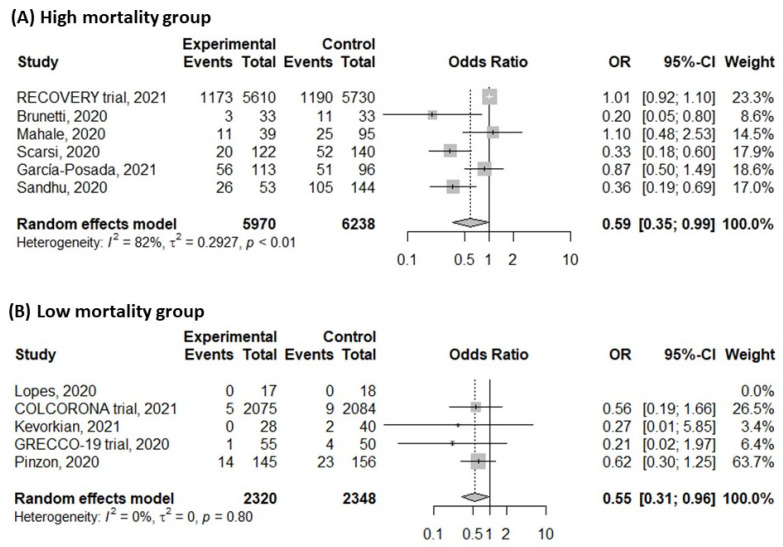
Forrest plot of mortality with subgroup analysis for mortality rate in colchicine group and control group. (**A**) High mortality group; (**B**) low mortality group.

**Figure 5:**
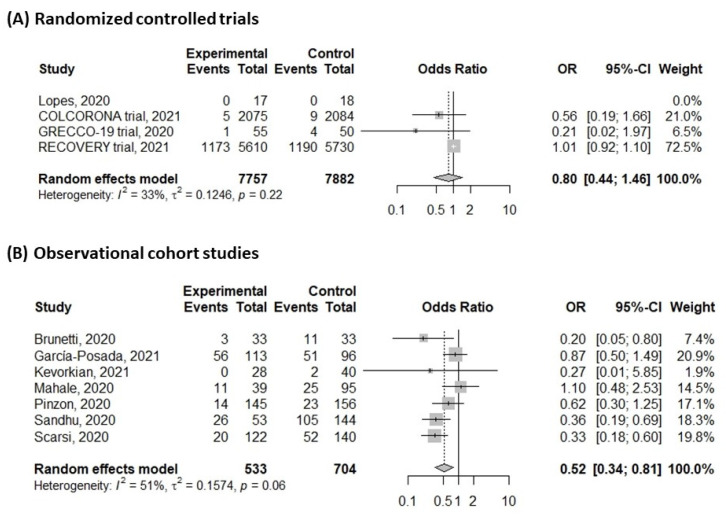
Forrest plot of mortality with subgroup analysis for randomized controlled trials in colchicine group and control group. (**A**) Randomized controlled trial; (**B**) observational cohort studies.

For assessing the risk of subsequent mortality, patients with colchicine treatment had a significantly lower risk of mortality with moderate heterogeneity (Figure 3) (OR: 0.57, 95% confidence interval (CI): 0.38–0.87, *I*^2^: 72%; *p* < 0.01). A sensitivity test was conducted, and the pooled estimates were not changed by individual trials. The funnel plot showed an asymmetric distribution of enrolled studies and was suggestive of publication bias (Supplementary File S2). Further contour-enhanced funnel plot and Egger’s test demonstrated the significance of publication bias (Supplementary Files S3 and S4; Egger’s test, *t* = −3.45, *p* = 0.0087). A wide range of overall mortality rates was observed, and the median mortality rate was approximate 20%. We defined the high and low mortality groups by more than 20% and less than 20%. Subgroup analysis with different mortality rate showed similar results (Figure 4) (high mortality group, OR: 0.59, 95%CI: 0.35–0.99, *I*^2^: 82%; *p* < 0.01; low mortality group, OR: 0.55, 95%CI: 0.31–0.96, *I*^2^: 0%; *p* = 0.8). A total of 10 studies investigating hospitalized patients and meta-analysis of these studies showed similar results (OR: 0.57, 95%CI: 0.37–0.89, *I*^2^: 75%; *p* < 0.01, Supplementary File S5). However, subgroup analyses with study type showed discrepant results; subgroup analysis with randomized controlled trials showed no significant differences between colchicine-treated and control groups (Figure 5A) (OR: 0.80, 95%CI: 0.44–1.46, *I*^2^: 33%; *p* = 0.22). However, subgroup analysis with cohort studies showed significantly lower risk in colchicine-treated group (Figure 5B) (OR: 0.52, 95%CI: 0.34–0.81, *I*^2^: 51%; *p* = 0.06). Funnel plots of subgroups with high mortality studies and randomized controlled trials were plotted, and certain asymmetry was observed (Supplementary Files S6 and S7).

### 3.3. Meta-Analysis of Secondary Outcomes

**Figure 6:**
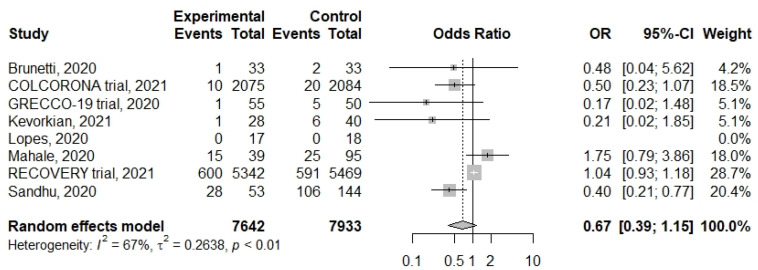
Forrest plot of risk for subsequent mechanical ventilation in colchicine group and control group.

A lower rate of subsequent mechanical ventilation was also observed in patients with colchicine treatment without statistical significance (Figure 6) (OR: 0.67, 95%CI: 0.39–1.15, *I*^2^: 67%; *p* < 0.01). Finally, the side effects were mild and not significantly different (Supplementary File S8) (OR; 2.03, 95%CI: 0.51–8.09, *I*^2^: 72%; *p* < 0.01).

## 4. Discussion

Based on our systematic review and meta-analysis, there was a significant improvement in mortality in the colchicine treatment group (OR: 0.57). Subgroup analysis with high and low mortality groups showed similar results. However, subgroup analysis with randomized controlled trials showed no statistically significant difference; thus, further studies are required to clarify the benefits of colchicine treatment in COVID-19.

The situation of COVID-19 varied in different countries and times [1,2,33]. The effects of intervention might differ in patients with different severity. For example, remdesivir was effective in patients requiring oxygen but no mechanical ventilation [8]. In our review, one study [17] enrolled non-hospitalized patients, and others were hospitalized. Meta-analysis of hospitalized patients showed a reduced risk of colchicine treatment (Supplementary File S5), and further studies were required to investigate the effects of colchicine on outpatients. A wide range of mortality rates was observed in our systematic review (0–72.9%), which was consistent with previous reports. We performed subgroup analysis to analyze the colchicine effect in the high and low mortality groups (Figure 4). Although the heterogeneity was low in the low mortality group and high in the high mortality group (high mortality group, *I*^2^: 82%; low mortality group, *I*^2^: 0%), colchicine was effective in both groups. Furthermore, although the quality of cohort studies was suitable, the strength of evidence was stronger for randomized controlled trials. We performed a subgroup analysis of randomized controlled trials and found no statistically significant benefits of colchicine (Figure 5). Therefore, we would not recommend routine colchicine treatment based on present evidence. Furthermore, asymmetry of funnel plots in both overall studies and randomized controlled trials was observed, and publication bias was suggested. Further contour-enhanced funnel plot and Egger’s test revealed the presence of significant publication bias. Various factors may contribute to the detected publication bias, including trials with negative or inconclusive results and outcome-reporting bias. Further, well-designed randomized controlled trials with appropriate randomization and comparison are warranted to elucidate the therapeutic role of colchicine on COVID-19.

In patients with COVID-19, multiple cytokine changes had been reported [5,34,35]. An evident elevation of pro-inflammatory cytokines, such as IL-6, was observed in patients with severe COVID-19 infection [6]. The inflammasome, a multiprotein complex of the innate immune responses, was activated in host responses to SARS-CoV-2 [34]. NLRP3-mediated inflammasome also played an important role in patients with severe COVID-19 [36,37,38]. Subsequent caspase 1-dependent release of the pro-inflammatory cytokines IL-1β and IL-18 followed, and gasdermin D-mediated pyroptotic cell death might occur. Furthermore, colchicine was found to be able to regulate NLRP3 inflammasome activation [39,40]. Colchicine use may disrupt this inflammatory pathway and inhibit subsequent systemic inflammatory disease, such as atherosclerosis [41]. Moreover, colchicine administration contributed to the reduction in IL-1β, IL-18, and IL-6 in patients with acute coronary syndrome [39]. These findings provide the theoretical basis for the clinical application of colchicine to combat COVID-19. Most authors in our review agreed with the benefits of colchicine treatment, and the meta-analysis found a significant reduction in mortality in colchicine treatment. Further subgroup analysis with randomized controlled trials showed no significant benefit. The complex underpinning immune-mediated mechanisms and interaction of colchicine and COVID-19 remained largely unclear.

Colchicine was an old drug, and physicians were familiar with its use [12]. Gastrointestinal adverse events and diarrhea were the commonly reported adverse events in our review. Discontinuation of trials due to colchicine-related adverse events was rare. The reported rates of adverse events were approximate 6% in our systematic review, and further meta-analysis showed a non-significant increase in colchicine treatment with moderate heterogeneity (OR; 2.03, 95%CI: 0.51–8.09, *I*^2^: 72%; *p* < 0.01). Differences in colchicine dose, frequency, and duration, and concomitant medication might contribute to the observed heterogeneity. In short, colchicine treatment was safe, and the adverse events were mild and not significantly different from the control group.

Our study was subjective to some limitations. First, the enrolled severity and timing of colchicine treatment were different in individual studies. The protocol of standard of care and concomitant medication differed in different hospitals and varied by time. For example, hydroxychloroquine was commonly used in the early pandemic but was seldom used after June 2020. Furthermore, although most studies investigated hospitalized patients and the subgroup analysis with different mortality rates were similar, different patient severity and quality of care might result in the observed wide range of mortality rates. These confounding factors might affect the effects of colchicine treatment and were reflected in the moderate heterogeneity (*I*^2^: 72%). The funnel plot and Egger’s test demonstrated the significant publication bias. Further high-quality randomized controlled studies were warranted to investigate the entire impacts of colchicine treatment and determine the optimal dose, interval, timing, and patients. Second, a comparison of laboratory tests may provide evidence of colchicine treatment, especially inflammatory markers. However, detailed laboratory tests were lacking in most studies. Finally, the enrolled patients were all adults, and most were elderly people; the effects of colchicine treatment might be different in children and adolescents.

## 5. Conclusions

In conclusion, our systematic review and meta-analysis identified 17,205 COVID-19 patients, and we found that a significant reduction in mortality in patients with colchicine treatment (OR: 0.57). The results were similar with subgroup analysis for different mortality rates. However, moderate heterogeneity was observed, and the dose, interval, duration, and mortality rate varied across studies. Further subgroup analysis with randomized controlled trials showed a non-significant decrease. Funnel plots and Egger’s test demonstrated a significant publication bias in both meta-analysis of all studies and randomized controlled trials. Therefore, further well-designed randomized controlled trials were required to elucidate the benefits of colchicine treatment and determine the optimal regimen. Although colchicine was cheap, easily available, accessible, and safe, routine colchicine treatment was not recommended based on our systematic review and meta-analysis.

## Figures and Tables

**Table 1:** Demographic characteristics of enrolled studies investigating colchicine in treating COVID-19.

Study, Year [Ref]	Country	Participants, N	Male, N (%)	Median Age (Years Old)	Severity	Mortality (Overall/Colchicine/Control, %)	Dose/Duration	Study Design	Concomitant Medication	Primary Outcomes	Secondary Outcomes	Author Conclusion *
**Brunetti, 2020 [24]**	USA	66	43 (65)	61.7	Hospitalized	21.2/9.1/33.3	1.2 mg followed by 0.6 mg 1 h later	Propensity-matched study	HCQ, AZI, tocilizumab, REM	28-day mortality	Clinical improvement, oxygen weaning, discharge	Y
**COLCORONA trial, 2021 [17]**	Brazil, Canada, Greece, South Africa, Spain, and the USA	4488	2069 (46.1)	53	Non-hospitalized	0.3/0.2/0.4	0.5 mg twice per day for 3 days and then once per day for 27 days thereafter	Phase 3 randomized, double-blinded trial	HCQ, anticoagulant, aspirin, other platelet agents	Mortality or hospital admission for COVID-19 30 days after randomization	Mechanical ventilation, pneumonias, adverse events	N (for all cases); Y (for PCR-confirmed cases)
**García-Posada, 2021 [26]**	Colombia	209	127 (61)	60	Hospitalized (100 in ICU)	51.2/49.6/53.1	20 days if no intolerance or hypersensitivity	Descriptive observational study	Antibiotics, low molecular weight heparin, corticosteroids, tocilizumab	Mortality	Clinical manifestations	Y
**GRECCO-19 trial, 2020 [25]**	Greece	105	61 (58.1)	63	Hospitalized	4.8/1.8/8	1.5 mg loading dose followed by 0.5 mg after 60 min and maintenance doses of 0.5 mg twice daily, 3 weeks	Prospective, open-label, randomized clinical trial	HCQ, AZI, Lopinavir or ritonavir, tocilizumab, anticoagulation	Maximum high-sensitivity cardiac troponin level; time for C-reactive protein increase and clinical deterioration	Mechanical ventilation; all-cause mortality; adverse events	Y (narrow margin of clinical significance)
**Kevorkian, 2021 [27]**	France	68	53 (77.9)	68	Hospitalized	2.9/0/5	1 mg followed by 0.5 mg 1 h later, then 0.5 mg q8 h for total 8 mg	Observational cohort study	Prednisolone, furosemide, salicylate, direct anti-Xa inhibitor	Oxygen use; mechanical ventilation; 28-day mortality	Adverse events	Y
**Lopes, 2020 [28]**	Brazil	35	14 (40)	48	Hospitalized (moderate to severe cases)	0/0/0	0.5 mg twice daily for 5 days, then 0.5 mg twice daily for 5 days	Randomized, double-blinded, placebo-controlled clinical trial	HCQ, AZI, heparin, methylprednisolone	Oxygen use; time of hospitalization; intensive care unit; death rate; and causes of mortality	Laboratory tests; adverse events, etc.	Y
**Mahale, 2020 [29]**	India	134	91 (68)	55.6	Hospitalized patients with oxygen therapy	26.9/28.2/26.3	0.5 mg/day for 1 week	Retrospective observational study	HCQ, AZI, methylprednisolone, etoricoxib, tocilizumab, Abx	In-hospital mortality	Clinical manifestations	ND
**Pinzón, 2020 [30]**	Colombia	301	178 (59.1)	56.8	Hospitalized for COVID-19 pneumonia	12.3/9.7/14.7	0.5 mg every 12 h for 7 to 14 days	Observational study	HCQ, AZI, corticosteroid, lopinavir/ritonavir, Abx	Mortality	Cormobidities, clinical manifestations	Y
**RECOVERY trial, 2021 [16]**	U.K. (Indonesia, Nepal)	11,340	7908 (69.7)	63.4	Hospitalized	20.8/20.9/20.8	1 mg followed by 0.5 mg 12 h later and then 0.5 mg twice for 10 days	Randomized, controlled, open-label trial	Dexamethasone, HCQ, AZI, lopinavir-ritonavir, tocilizumab, and convalescent plasma	28-day all-cause mortality	Discharge; mechanical ventilation	N
**Sandhu, 2020 [31]**	USA	197	114 (57.9)	70	Hospitalized (moderate to severe)	66.5/49.1/72.9	0.6 mg twice a day for three days and then 0.6 mg once a day (total 12 days)	Prospective comparative cohort study (case control)	HCQ, steroid, enoxaparin, heparin, etc.	Mortality, mechanical ventilation	Inflammatory markers	Y
**Scarsi, 2020 [32]**	Italy	262	167 (63.7)	69.3	Hospitalized, with pneumonia	27.5/16.4/37.1	1 mg/day	Prospective cohort study	HCQ, dexamethasone, lopinavir/ritonavir	Mortality	Clinical manifestations	Y

* Y: Authors support colchicine use; N: No significant difference between colchicine and control group; ND; not described; # Abbreviations: Abx: antibiotics; AZI, azithromycin; HCQ: hydroxychloroquine; REF: reference; REM: remdemsivir.

## Supplementary Materials

The following are available online at https://www.mdpi.com/article/10.3390/life11080864/s1, Supplementary File S1, Complete search strategy of our systematic review. Supplementary File S2, Funnel plot of enrolled studies investigating the subsequent mortality of colchicine and control groups. Supplementary File S3, Contour-enhanced funnel plot of enrolled studies investigating subsequent mortality of colchicine and control groups. Supplementary File S4, Egger’s test of enrolled studies investigating subsequent mortality of colchicine and control groups. Supplementary File S5, Forest plot of enrolled studies investigating the subsequent mortality of colchicine and control groups in hospitalized patients. Supplementary File S6, Funnel plot of enrolled studies investigating the subsequent mortality of colchicine and control groups in studies with high mortality rates. Supplementary File S7, Funnel plot of enrolled studies investigating the subsequent mortality of colchicine and control groups in randomized controlled trials. Supplementary File S8, Forest plot of enrolled studies investigating the adverse events of colchicine and control groups.
