# Supplementary material for: Repurposing Colchicine in Treating Patients with COVID-19: A Systematic Review and Meta-Analysis
Source: Life (Basel). 2021 Aug 23;11(8):864. doi: 10.3390/life11080864 (PMC8398430; doi:10.3390/life11080864)
Supplement: Supplementary file 1 [file life-11-00864-s001.zip › Supplementary File S1, Complete search strategy of our systematic review..pdf]

Supplementary File S1. Complete search strategy of our systematic review.

Pubmed
Search Strategy:
1 (covid 19 or covid-19).
2 "coronavirus disease 2019"
3 SARS-CoV-2
4 or/1-3
5 exp Colchicine
6 colchicine
7 or/5-6
8 4 and 7

Embase
Search Strategy:
1 (covid 19 or covid-19)
2 "coronavirus disease 2019"
3 SARS-CoV-2
4 or/1-3
5 exp Colchicine
6 colchicine
7 or/5-9
8 4 and 7

----

medRxiv
covid 19 AND (colchicine)

Research Square
covid 19 AND colchicine
