# Supplementary figures and images for: Repurposing Colchicine in Treating Patients with COVID-19: A Systematic Review and Meta-Analysis
Source: Life (Basel). 2021 Aug 23;11(8):864. doi: 10.3390/life11080864 (PMC8398430; doi:10.3390/life11080864)

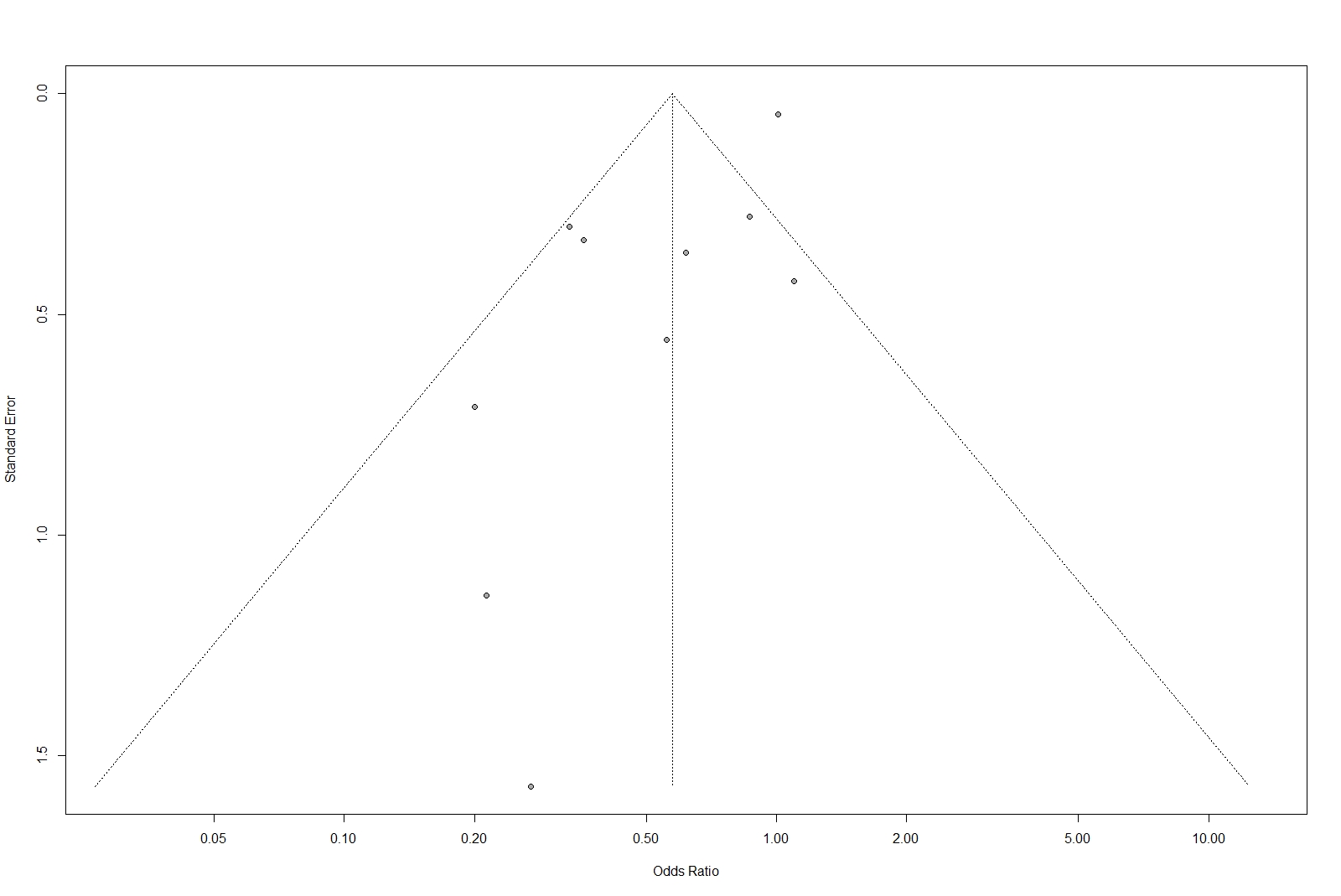

Supplement: Supplementary file 1 [file life-11-00864-s001.zip › Supplementary File S2, Funnel plot of enrolled studies investigating the subsequent mortality of colchicine and control groups..jpeg]

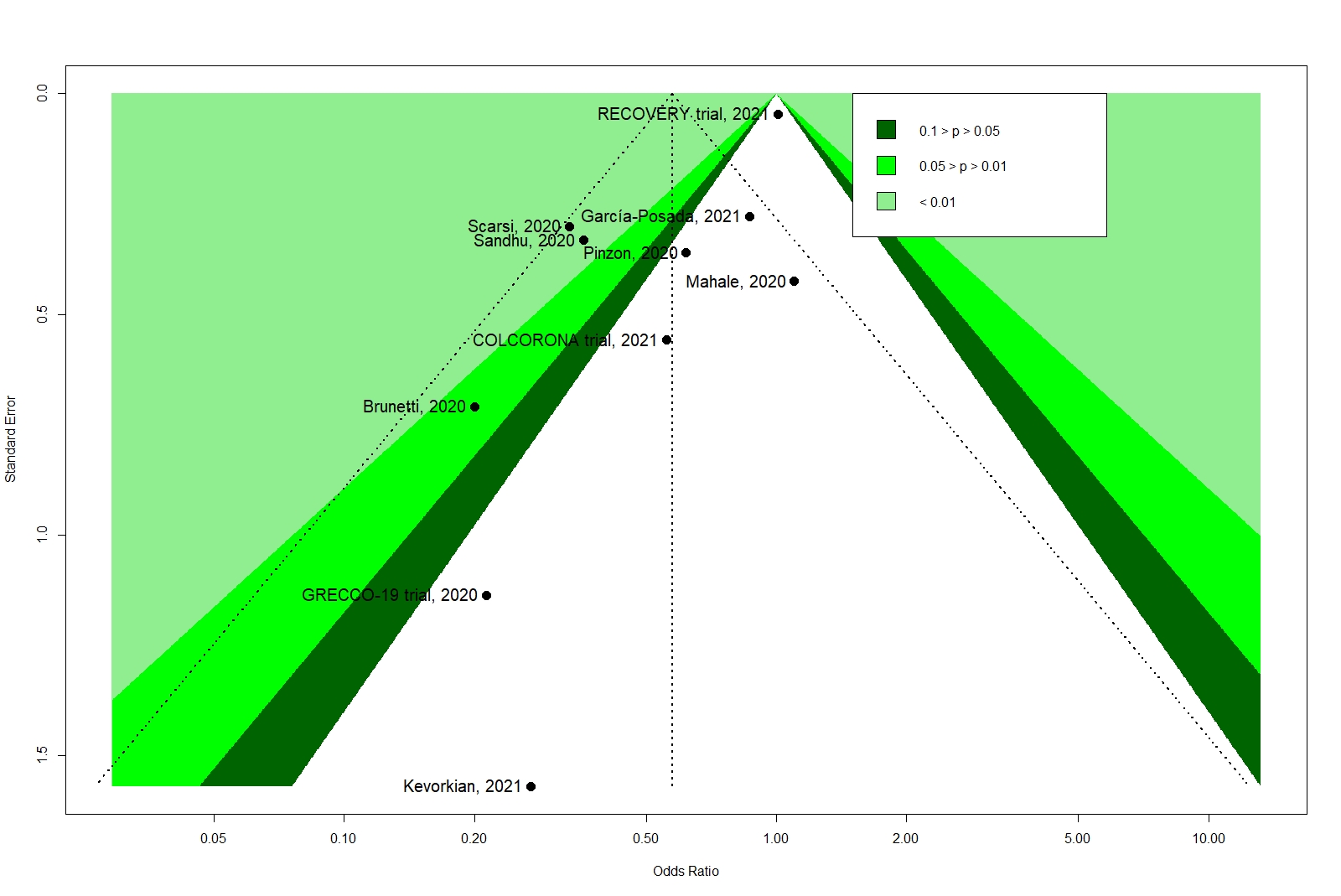

Supplement: Supplementary file 1 [file life-11-00864-s001.zip › Supplementary File S3, Contour-enhanced funnel plot of enrolled studies investigating subsequent mortality of colchicine and control groups..jpeg]

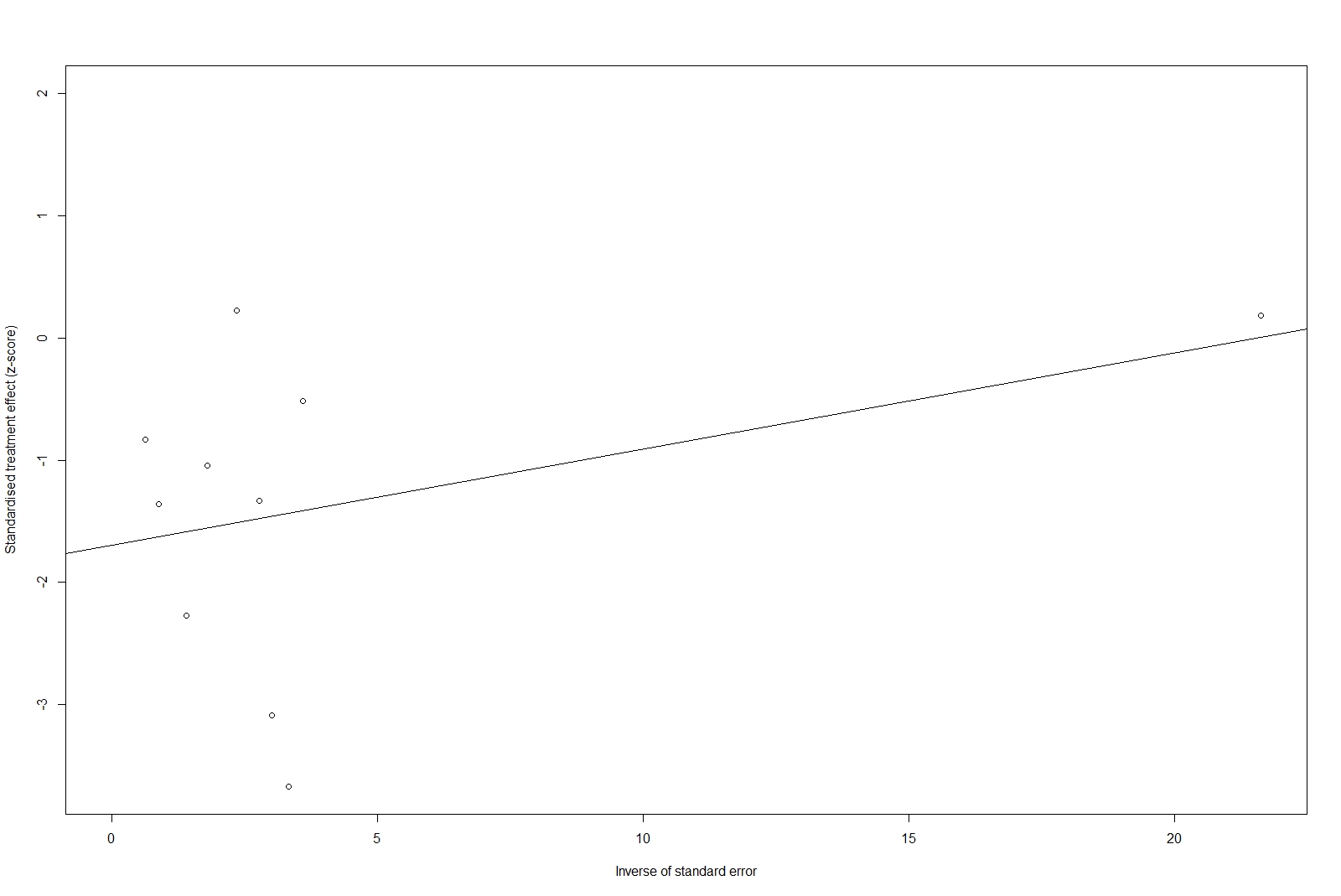

Supplement: Supplementary file 1 [file life-11-00864-s001.zip › Supplementary File S4, Eggerí»s test of enrolled studies investigating subsequent mortality of col-chicine and control groups..jpeg]

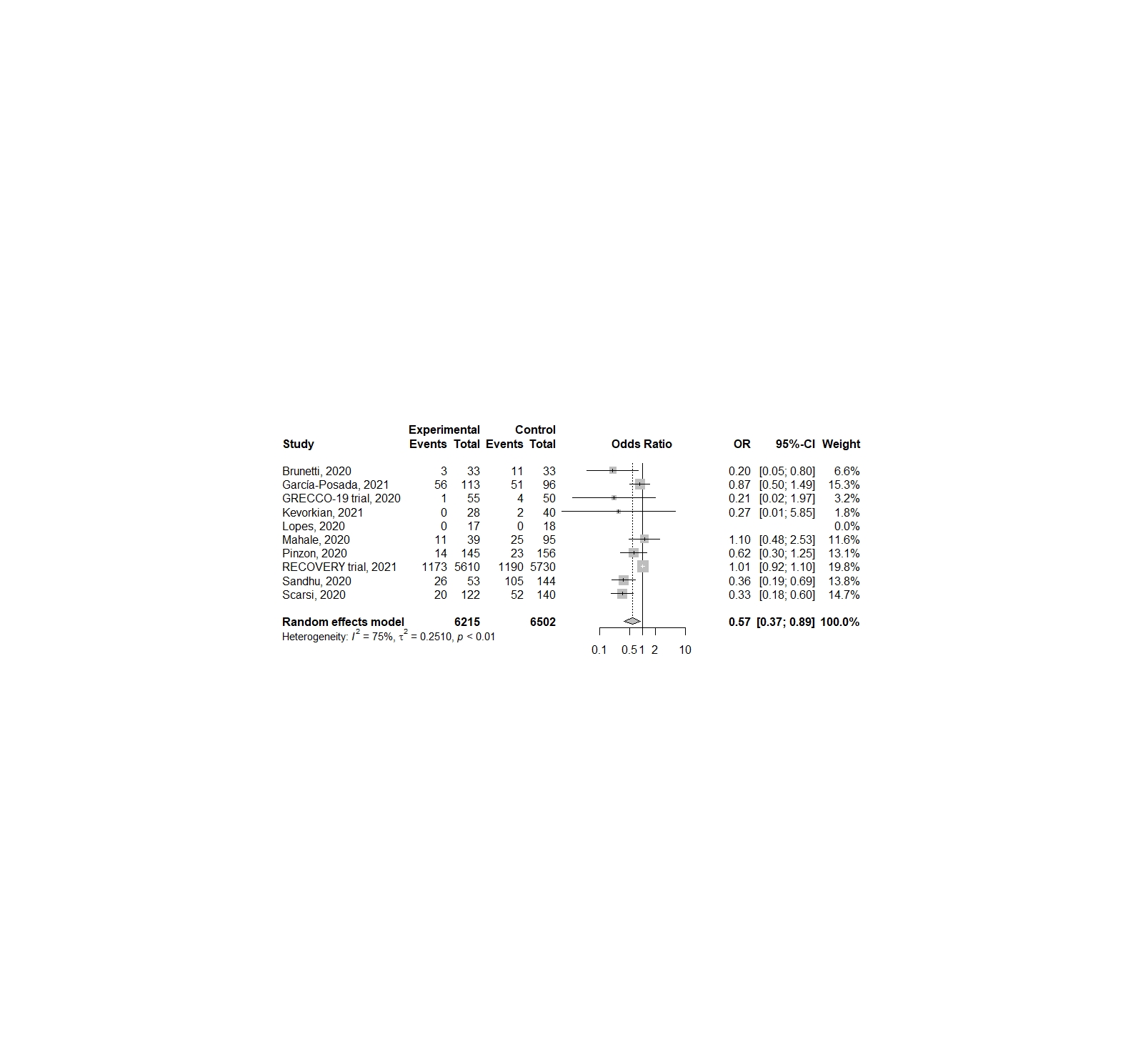

Supplement: Supplementary file 1 [file life-11-00864-s001.zip › Supplementary File S5, Forest plot of enrolled studies investigating the subsequent mortality of colchicine and control groups in hospitalized patients..jpeg]

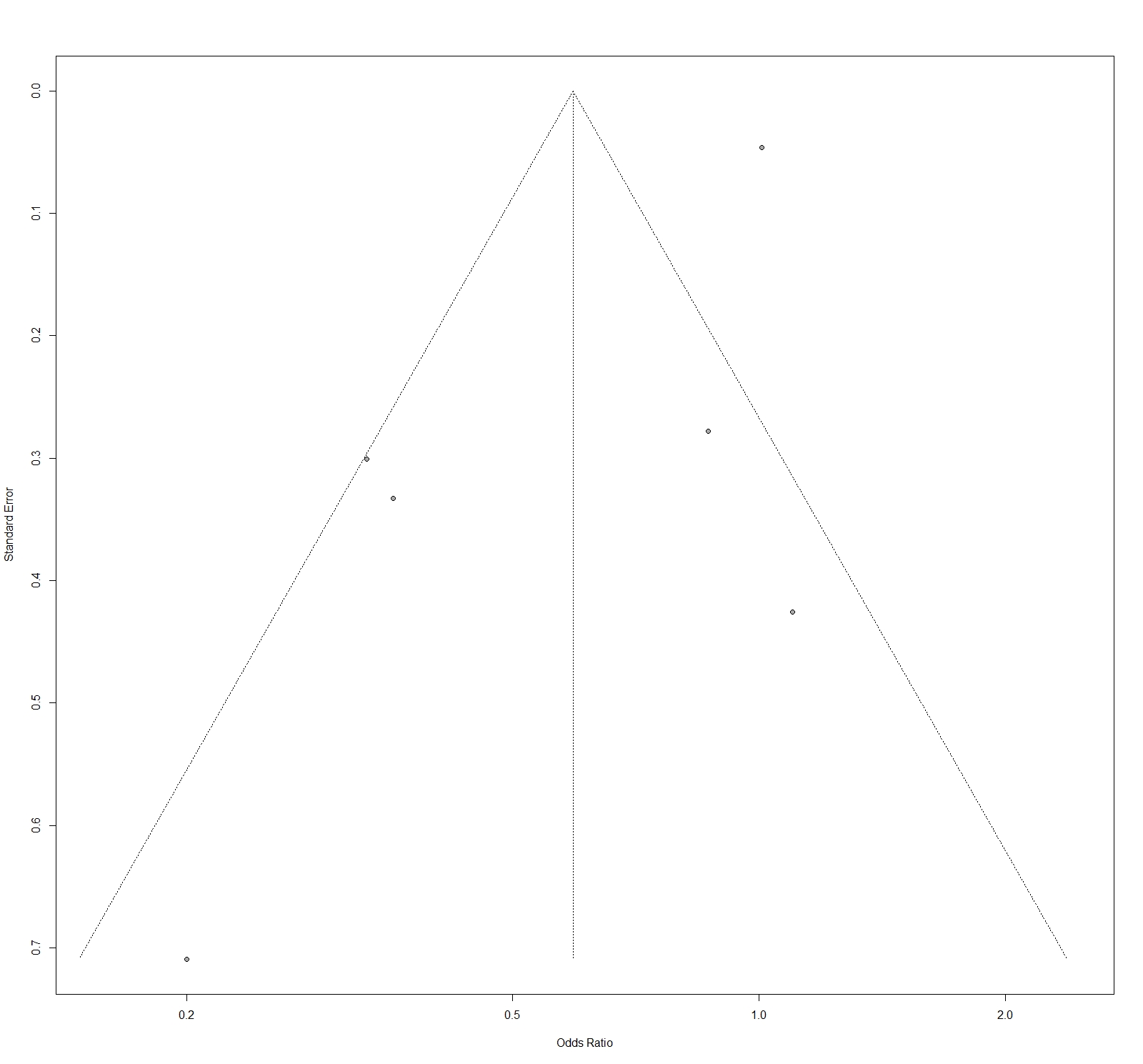

Supplement: Supplementary file 1 [file life-11-00864-s001.zip › Supplementary File S6, Funnel plot of enrolled studies investigating the subsequent mortality of colchicine and control groups in studies with high mortality rates..jpeg]

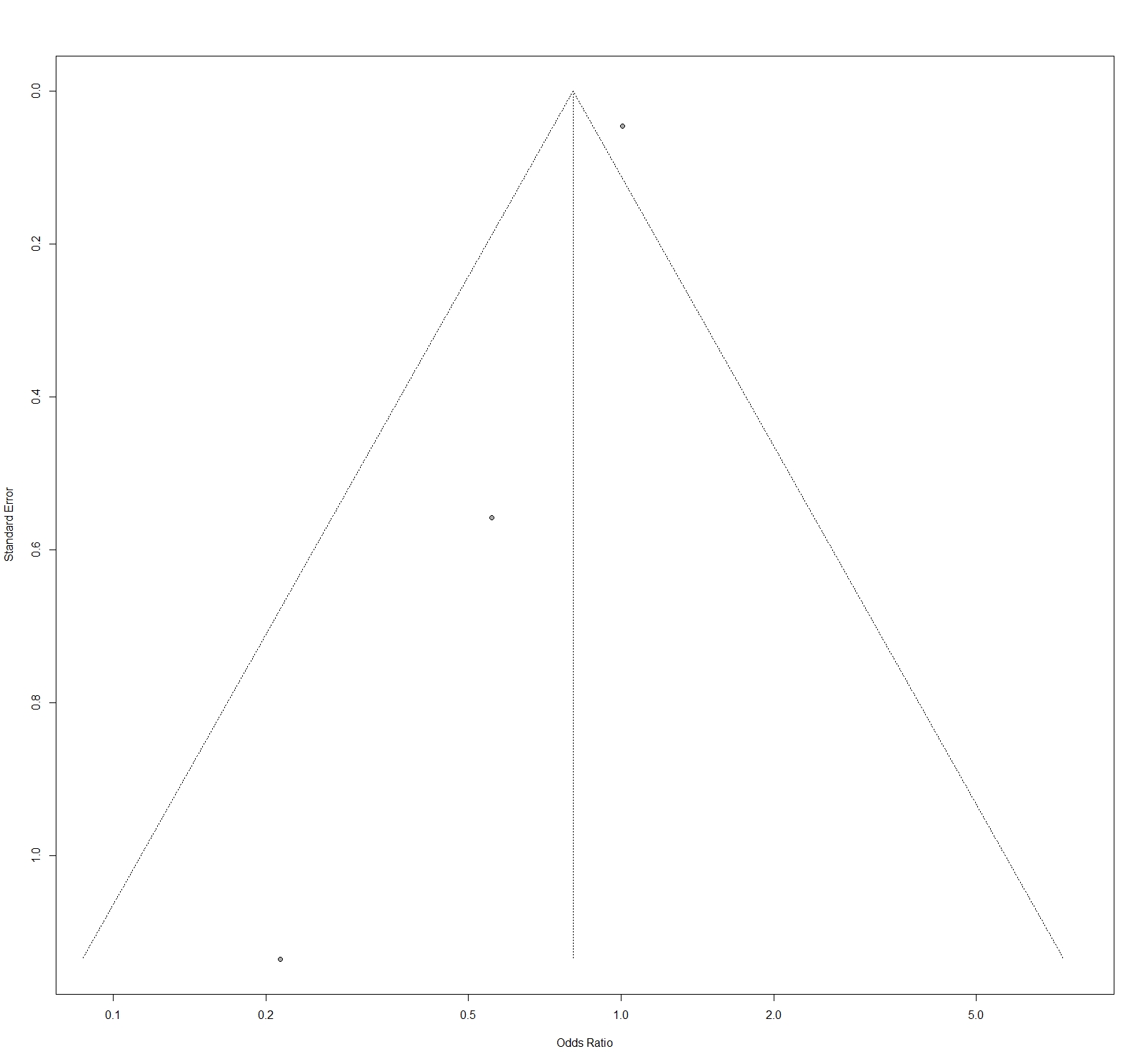

Supplement: Supplementary file 1 [file life-11-00864-s001.zip › Supplementary File S7, Funnel plot of enrolled studies investigating the subsequent mortality of colchicine and control groups in randomized controlled trials..jpeg]

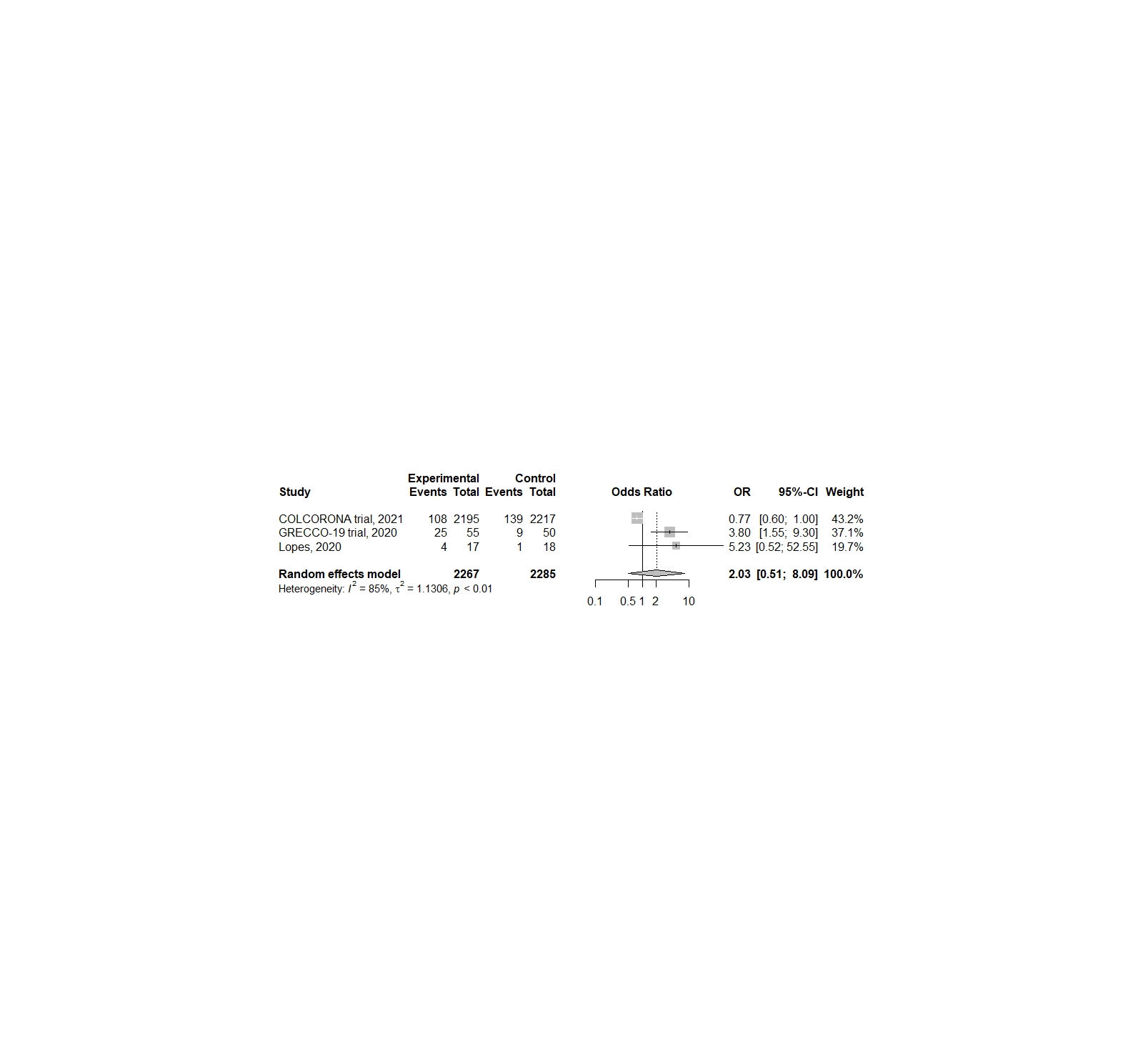

Supplement: Supplementary file 1 [file life-11-00864-s001.zip › Supplementary File S8, Forest plot of enrolled studies investigating the adverse events of colchi-cine and control groups..jpeg]
